# Maternal Anemia in Rural Jordan: Room for Improvement

**DOI:** 10.1155/2011/381812

**Published:** 2011-09-12

**Authors:** Lama Al-Mehaisen, Yousef Khader, Oqba Al-Kuran, Fayrouz Abu Issa, Zouhair Amarin

**Affiliations:** ^1^Deprtment of Obstetrics and Gynecology, Faculty of Medicine, Jordan University of Science and Technology, P.O. B 630017, Irbid 22110, Jordan; ^2^Department of Public Health, Community Medicine, and Family Medicine, Faculty of Medicine, Jordan University of Science and Technology, P.O. B 630017, Irbid 22110, Jordan

## Abstract

The objectives of this cross-sectional study were to estimate the prevalence and determine factors associated with anemia among pregnant women in rural Jordan. A cohort of 700 pregnant women from a National Health Service hospital and ten health centers completed a questionnaire. Of the total, 243 (34.7%) had anemia. The prevalence was the highest for women in their 3rd trimester (42.5%) compared to those in 2nd trimester (32.7%) and 1st trimester (18.9%). Gestational age, body mass index, history of previous surgery, and multivitamin intake during pregnancy were significantly associated with anemia. Women in the 2nd and 3rd trimesters had higher odds of anemia (OR = 2.2 and 3.3, resp.). Underweight women had higher odds of anemia (OR = 2.9). History of previous surgery and multivitamin intake during pregnancy were associated with higher odds of anemia (OR = 1.6 and 1.9, resp.).

## 1. Introduction

Reducing maternal mortality is one of the eight health related Millennium Development Goals (MDG5) adopted at the Millennium Summit in 2000. Within this framework, the international community is committed to reduce Maternal Mortality by three quarters between 1990 and 2015 [1]. Hemorrhage is the leading cause of maternal mortality [2]. Anemia is one of the world's leading causes of hemorrhage and disability [3] and thus is one of the most serious global public health problems.

The impact of anemia in pregnancy affects both mother and fetus [4–13]. Anemia in pregnancy is one of the predisposing factors for preterm delivery [5, 12–15], low birth weight [6–16], stillbirth and neonatal death [7, 8] and maternal deaths [17, 18]. 

Anemia is one of the most prevalent nutritional deficiency problems affecting pregnant women [19]. The prevalence of anemia in pregnancy varies considerably because of differences in socioeconomic conditions, lifestyles, and health-seeking behaviors across different cultures [20–32]. The World Health Organization (WHO) estimates that 52% of pregnant women in developing countries are anemic compared with 23% in the developed world [3].

Factors associated with anemia among pregnant women identified in past studies included parasite infestation [33–37], season [38], dietary habits [37–39], gestational age [30–39], parity [25, 27, 39], gravidity [22], age at the time of marriage, geographic location [36, 39], interval between pregnancies [25, 30], educational level [30, 40], and smoking [31].

Because anemia is the most frequent maternal complication of pregnancy, antenatal care should be concerned with its early detection and management [15]. The objective of this study was to estimate the prevalence of anemia amongst pregnant women in rural Jordan and to determine its associated factors (see Table 5).

## 2. Methods

This cross-sectional study was conducted among pregnant women in Bani-Kenana rural district in the period between April and August 2009. A total of 700 consecutive pregnant women who attended the main hospital in Bani-Kenana, a rural area in the north of Jordan, for antenatal care during the study period were invited to participate in the study. All women agreed to participate. An informed verbal consent was obtained from each woman. All women had spontaneous pregnancies. 

The study was approved by the Institutional Review Board of Jordan University of Science and Technology. A questionnaire was filled in by a trained midwife. The questionnaire sought information about sociodemographic characteristics that included age, educational level for women and their husbands, occupation, and monthly income. Data about obstetrical characteristics included gravidity, parity, interpregnancy interval (defined as the time in months between the woman's last delivery and the date of the last menstrual period for the index pregnancy), and previous mode of delivery. Medical conditions such as diabetes, renal disease, hypertension, previous surgeries, hemorrhoids, and peptic ulcer disease were documented. 

As for the current pregnancy, last menstrual period, use of iron, use of folic acid and multi-vitamin intake were noted. Prepregnancy weight and height were self-reported. Body Mass Index (BMI) was calculated. Women were then categorized into four groups according to their BMI as follows: underweight (less than or equal to a BMI of 19.9 kg/m^2^), normal (BMI of 20–24.9 kg/m^2^), overweight (BMI of 25–29.9 kg/m^2^), and obese (BMI ≥ 30–34.9 kg/m^2^). The group with BMI in the normal range (20–24.9 kg/m^2^) was used as the reference or comparison group for the analysis.

Hemoglobin value of less than 11.0 g/dL was used to define anemia in pregnancy. The degrees of anemia studied were mild anemia (hemoglobin 9.0–10.9 g/dL), moderate anemia (hemoglobin 7.0–8.9 g/dL), and severe anemia (hemoglobin less than 7.0 g/dL). Venous blood samples were obtained and analyzed for complete blood count using a standardized hematological screen at the hospital's laboratory.

Statistical analysis was carried out using the Statistical Package for Social Sciences Software (SPSS version 15). Descriptive statistics were obtained using means and percentages. Univariate analysis of the differences in the prevalence of anemia according to relevant characteristics was analyzed using chi-square test. Multivariate analysis using binary logistic regression was conducted to identify factors associated with anemia. A *P* value of less than 0.05 was considered statistically significant.

## 3. Results

Table 1 summarizes the sociodemographic, obstetric, and relevant characteristics of women. Their age ranged from 15 to 45 years with a mean (SD) of 28.8 (5.8) years. About half of women (43.4%) were 30 years of age or more. Only one third had educational level higher than high school and 13.7% were employed. 

Of the total of 700 pregnant women, 243 (34.7%) had anemia (0.1% severe anemia, 2.9% moderate anemia, and 31.7% mild anemia). The prevalence was the highest for women in their 3rd trimester (42.5%) compared to those in the 2nd trimester (32.7%) and the 1st trimester (18.9%). The prevalence of anemia according to sociodemographic, obstetric, and relevant characteristics of women is shown in Tables 2, 3 and 4. 

In multivariate analysis, the only variables that were significantly associated with anemia were gestational age, BMI, previous surgery, and multi-vitamin intake during pregnancy. 

Compared to women in their 1st trimester, women in the 2nd and 3rd trimesters had higher odds of anemia (OR = 2.2 and 3.3, resp.). Compared to women with normal BMI, underweight women had higher odds of anemia (OR = 2.9). History of previous surgery and multi-vitamin intake during pregnancy were associated with higher odds of anemia (OR = 1.6 and 1.9, resp.).

## 4. Discussion

In Jordan, among 1406 deaths of women of reproductive age identified for the 2007–2008 period out of 397 588 live births, 76 (5.4%) maternal deaths were identified, giving an MMR of 19.1 deaths per 100 000 live births. Of the 76 maternal deaths, 19 (25.0%) were caused by hemorrhage, which was the most common direct cause of maternal death and the most frequent cause-specific maternal mortality factor [41]. 

In this study, the prevalence of anemia among pregnant women was 34.7%. Prevalence of anemia in pregnancy varies from country to another and from region to another. Previous studies reported that the prevalence of anemia among pregnant women was 20.1% (hemoglobin < 11.0 g/dL in the first and third trimesters or < 10.5 g/dL in the second trimester of pregnancy) in Thailand [19], 13% (Anemia was defined using the 5th percentile cutoff for each week of gestational age as proposed by Yip from the Centers of Disease Control, 1989) in Puente Alto, Chile [20], 30.2% (hemoglobin < 10.5 g/dL) in Korea [21], 19.7% in South Africa [22], 34.4% (hemoglobin < 11 g/dL) in Venezuela [23], 35.3% (hemoglobin < 11 g/dL) in Lagos, Nigeria [24], 70.1% in Peru [25], 41.9% in Jima town Ethiopia [26], 55% (hemoglobin < 11 gm/dL) in Sharkia Governorate, Egypt [27], 25.8% in North of Iran [28], 31.9% (hemoglobin < 11 g/dL) in Asir region, Saudi Arabia [29], 16.7% (hemoglobin < 11 g/dL) in Southern Iran [30], and 42% in Northern Jordan [31].

The prevalence of anemia in this study increased with increased trimester of pregnancy. This finding is consistent with the findings of Haniff et al. [39], where most anemic cases were found to be in the second and third trimesters. This may be due to an increased demand for micronutrients during the last trimester. 

Underweight women had higher odds of anemia compared to women with normal BMI. This finding is consistent with the finding of Hollander [41] and Mardones et al. [19]. This supports the concept that pregnant women with low BMI need special attention for prevention and treatment of anemia.

In Jordan, the standard multivitamin tablets contain iron. Of women who were taking multivitamins, 36.6% were anemic. No details were available regarding pre-treatment levels. It is speculated that women with anemia were more likely to be prescribed multivitamins. In a study from Tanzania by Makola et al. [42], after 8 weeks of supplementation, the risk of anemia during pregnancy was reduced by 51% and the risk of iron deficiency anemia by 56%. The supplement reduced the risk of becoming iron deficient or developing iron deficiency by 53% and increased Hb concentration by 4.16 g/dL.

Maternal age, gravidity, and parity were not significantly associated with anemia. This is consistent with the findings of Desalegn et al. in which parity was not a significant predictor of Hb concentration [25]. Similarly, Dim and Onah found that maternal age and parity had no statistical relationship with the prevalence of anemia at booking in a Nigerian population [43]. 

In this study, the prevalence of anemia was not affected by age. This is contrary to the findings of Mahfouz et al. who found that the prevalence of anemia in Saudi women was highest among those who were less than 20 years old [28]. In addition, no significant association was found between anemia and low socioeconomic and educational levels. This differs from other studies in which women from lower socioeconomic class had higher risk for developing anemia in pregnancy [44, 45]. 

Regarding Interpregnancy intervals, our results showed that there was no significant association with anemia. This finding is in contradiction with that obtained by other authors [26]. It may be speculated that women who manage to get pregnant are those with a relatively better hematologic indices, and those with severe anemia are less likely to ovulate and get pregnant. Further studies addressing this particular parameter are required. 

This study found no significant association between chronic medical illnesses and anemia in pregnancy. This is in agreement with the findings of Lieberman et al. where hemolysis, renal, hepatic, and pulmonary disorders were less common causes of anemia in pregnant women [13]. 

In this study, tea consumption was not associated with anemia. This is in disagreement with the findings of other studies in which tea consumption was associated with anemia, as it may interfere with iron absorption. This controversy needs further investigation. 

One of the limitations of this study was its cross-sectional nature and reliance on retrospectively reviewing patients' case notes. Followup of pregnancies for potential complications would have increased the scientific merit of this study.

It is concluded that the prevalence of anemia among pregnant women is still high in certain areas. In the commitment to reduce the maternal mortality by three-quarters between 1990 and 2015 efforts should be geared towards early detection and treatment of anemia, and it is recommended that health education should be provided to mothers on the need for medical evaluation before pregnancy. Pregnant women should be screened for anemia. Screening should be undertaken early in pregnancy.

## Figures and Tables

**Table 1:** Sociodemographic, obstetric, and relevant characteristics.

Variables	*n* (%)
Age (year)	
<25	187 (26.7)
25–29.9	209 (29.9)
≥30	304 (43.4)
Education level	
≤High school	473 (67.6)
>High school	227 (32.4)
Income/per month (in Jordanian dinars, 1 JD = $1.41)	
≤250	354 (50.6)
>250	346 (49.4)
Employment	
Yes	96 (13.7)
No	603 (86.3)
Smoker	16 (2.3)
Body mass index (kg/m²)	
Underweight (<19.8)	16 (2.3)
Normal (19.8–26)	243 (34.7)
Overweight (26–29)	167 (23.9)
Obese (>29)	274 (39.1)
Parity	
Zero	190 (27.1)
One	139 (19.9)
Two	122 (17.4)
Three	87 (12.4)
≥four	162 (23.1)
Trimester	
1st	143 (20.4)
2nd	211 (30.1)
3rd	346 (49.4)
Interpregnancy interval/months	
1st pregnancy	175 (25.0)
<12	138 (19.7)
12–23.9	161 (23.0)
≥24	226 (32.3)
Presence of chronic diseases	25 (6.6)
Previous cesarean section	114 (16.3)
History of previous surgeries	196 (28)
Hemorrhoids	117 (16.7)
Peptic ulcer	27 (3.9)
Folic acid intake in this pregnancy	520 (74.3)
Multivitamins intake in this pregnancy	187 (26.7)
Ferrus sulphate intake in this pregnancy	399 (57)

**Table 2:** Prevalence of anemia according to sociodemographic characteristics.

Variable	Hb level (mg/dl)	Anemia		Total	*P* value
	Mean (SD)	No	Yes		
Age (year)					0.361
<25	11.3 (1.2)	120 (64.2)	67 (35.8)	178	
25–29.9	11.2 (1.2)	130 (62.2)	79 (37.8)	209	
≥30	11.5 (1.1)	207 (86.1)	97 (31.9)	304	
Number of family members					0.808
Two	11.3 (1.2)	111 (67.3)	54 (32.7)	165	
Three	11.3 (1.1)	86 (63.2)	50 (36.8)	136	
Four	11.4 (1.1)	85 (67.5)	41 (32.5)	126	
≥five	11.4 (1.1)	175 (64.1)	98 (35.9)	273	
Education level					0.135
≤high school	11.3 (1.2)	300 (63.4)	173 (36.6)	473	
>high school	11.4 (1.1)	157 (69.2)	70 (30.8)	227	
Husband education level					0.544
≤high school	11.4 (1.1)	348 (65.9)	180 (34.1)	528	
>high school	11.3 (1.3)	109 (63.4)	63 (36.6)	172	
Income/per month (in Jordanian dinars, 1 JD = $1.41)					0.646
≤250	11.4 (1.1)	234 (66.1)	120 (33.9)	354	
>250	11.3 (1.2)	223 (64.5)	123 (35.5)	346	
Employment					0.313
Yes	11.3 (1.1)	389 (64.5)	214 (35.5)	603	
No	11.5 (1.1)	67 (69.8)	29 (30.2)	96	
Smoking status of women					0.768
Yes	11.3 (1.1)	11 (68.8)	5 (31.3)	16	
No	11.4 (1.1)	446 (65.2)	238 (34.8)	684	
Smoking status of husband					0.950
Yes	11.4 (1.2)	249 (65.2)	133 (34.8)	382	
No	11.3 (1)	208 (65.4)	110 (34.6)	318	

**Table 3:** Prevalence of anemia according to obstetrical characteristics.

Variable	Hb level (mg/dl)	Anemia		Total	*P* value
	Mean (SD)	No	Yes		
Ferrus sulphate intake in this pregnancy					0.002
Yes	11.2 (1.2)	241 (60.4)	158 (39.6)	399	
No	11.6 (1)	261 (71.8)	85 (28.2)	301	
Gravidity					0.618
One	11.4 (1.2)	115 (68.9)	52 (31.1)	167	
Two	11.1 (1)	75 (60)	50 (40)	125	
Three	11.4 (1.1)	69 (63.9)	39 (36.1)	108	
Four	11.5 (1.1)	59 (66.3)	30 (33.7)	89	
≥five	11.4 (1.2)	139 (65.9)	72 (34.1)	211	
Parity					0.713
Zero	11.4 (1.2)	130 (68.4)	60 (31.6)	190	
One	11.3 (1.1)	85 (61.2)	54 (38.8)	139	
Two	11.4 (1.1)	81 (66.4)	41 (33.6)	122	
Three	11.4 (1.1)	57 (65.5)	30 (34.5)	87	
≥four	11.3 (1.2)	104 (64.5)	58 (35.5)	162	
Previous cesarean section					0.461
Yes	11.5 (1.3)	71 (62.3)	43 (37.7)	114	
No	11.3 (1.1)	386 (65.9)	200 (34.1)	586	
Trimester					<0.005
1st	12 (1.1)	116 (81.1)	27 (18.9)	143	
2nd	11.4 (1)	142 (67.3)	69 (32.7)	211	
3rd	11.1 (1.2)	199 (57.5)	147 (42.5)	346	
Interpregnancy interval/months					0.769
1st pregnancy	11.4 (1.2)	118 (67.4)	57 (32.6)	175	
<12	11.4 (1.2)	93 (67.4)	45 (32.6)	138	
12–23.9	11.4 (1.2)	102 (63.4)	59 (36.6)	161	
≥24	11.3 (1)	144 (63.7)	82 (36.3)	226	
Body mass index (kg/m²)					0.372
Underweight (<19.8)	11.3 (1.6)	8 (50)	8 (50)	16	
Normal (19.8–26)	11.3 (1.2)	154 (63.4)	89 (36.6)	243	
Overweight (26–29)	11.4 (1.1)	108 (64.7)	59 (35.3)	167	
Obese (>29)	11.4 (1.1)	187 (68.2)	87 (31.8)	274	

**Table 4:** Prevalence of anemia according to medical characteristics.

Variable	Hb level (mg/dl)	Anemia		Total	*P* value
	Mean (SD)	No	Yes		
Present of chronic diseases					0.772
Yes	11.6 (1.1)	17 (68)	8 (32)	25	
No	11.3 (1.1)	440 (65.2)	235 (34.8)	675	
History of previous surgeries					0.053
Yes	11.4 (1.2)	117 (59.7)	79 (40.3)	196	
No	11.3 (1.1)	340 (67.5)	164 (32.5)	504	
Hemorrhoids					0.935
Yes	11.5 (1.2)	76 (65)	41 (35)	117	
No	11.3 (1.1)	381 (65.4)	202 (34.6)	583	
Peptic ulcer					0.502
Yes	11.3 (1)	16 (59.3)	11 (40.7)	27	
No	11.4 (1.1)	441 (65.5)	232 (34.5)	673	
Folic acid intake in this pregnancy					0.037
Yes	11.3 (1.1)	328 (63.1)	192 (36.9)	520	
No	11.5 (1.2)	129 (71.7)	51 (28.3)	180	
Multi-vitamins intake in this pregnancy					<0.005
Yes	11 (1.3)	98 (52.4)	89 (47.6)	187	
No	11.5 (1.1)	359 (70)	154 (30)	513	

**Table 5:** Multivariate analysis of factors associated with anemia.

Variables	OR (95% CI)	*P* value
Trimester		
1st	1	
2nd	2.2 (1.3, 3.6)	0.004
3rd	3.3 (2.0, 5.5)	<0.005
Body mass index (kg/m²)		
Underweight	2.9 (1.02, 8.3)	0.044
Normal	1	
Overweight	0.8 (0.6, 1.3)	0.464
Obese	0.7 (0.5, 1.02)	0.068
History of previous surgeries		
Yes	1.6 (1.1, 2.2)	0.016
No	1	
Multi-vitamins intake in this pregnancy		
Yes	1.9 (1.3, 2.7)	<0.005
No	1	
